# Optimized Genetic Testing for Polledness in Multiple Breeds of Cattle

**DOI:** 10.1534/g3.119.400866

**Published:** 2019-11-25

**Authors:** Imtiaz A. S. Randhawa, Brian M. Burns, Michael R. McGowan, Laercio R. Porto-Neto, Ben J. Hayes, Ryan Ferretti, Karen M. Schutt, Russell E. Lyons

**Affiliations:** * School of Veterinary Science, University of Queensland, Gatton, QLD 4343, Australia,; † Department of Agriculture and Fisheries, Rockhampton, QLD 4702, Australia,; ‡ CSIRO Agriculture and Food, St Lucia, QLD 4067, Australia,; § Centre for Animal Science, Queensland Alliance for Agriculture and Food Innovation, University of Queensland, St Lucia, QLD 4072, Australia,; ** Bioinformatics and Biostatistics, Neogen GeneSeek, Lincoln, NE, 68504 and; †† Neogen Australasia, University of Queensland, Gatton, QLD 4343, Australia

**Keywords:** Poll gene, bovine, dehorning, genetic testing, animal welfare, Genomic Prediction, GenPred, Shared Data Resources

## Abstract

Many breeds of modern cattle are naturally horned, and for sound husbandry management reasons the calves frequently undergo procedures to physically remove the horns by disbudding or dehorning. These procedures are however a welfare concern. Selective breeding for polledness – absence of horns – has been effective in some cattle breeds but not in others (*Bos indicus* genotypes) due in part to the complex genetics of horn phenotype. To address this problem different approaches to genetic testing which provide accurate early-in-life prediction of horn phenotype have been evaluated, initially using microsatellites (MSAT) and more recently single nucleotide polymorphism (SNP). A direct gene test is not effective given the genetic heterogeneity and large-sized sequence variants associated with polledness in different breeds. The current study investigated 39,943 animals of multiple breeds to assess the accuracy of available poll testing assays. While the standard SNP-based test was an improvement on the earlier MSAT haplotyping method, 1999 (9.69%) out of 20,636 animals tested with this SNP-based assay did not predict a genotype, most commonly associated with the Indicus-influenced breeds. The current study has developed an optimized poll gene test that resolved the vast majority of these 1999 unresolved animals, while the predicted genotypes of those previously resolved remained unchanged. Hence the optimized poll test successfully predicted a genotype in 99.96% of samples assessed. We demonstrated that a robust set of 5 SNPs can effectively determine P_C_ and P_F_ alleles and eliminate the ambiguous and undetermined results of poll gene testing previously identified as an issue in cattle.

Cattle, like other bovidae species, were historically naturally horned with bony appendages fused onto the frontal cranium for defense from predators and to exert dominance for food and mating (Kiltie 1985; Lundrigan 1996; Davis *et al.* 2011). Since domestication of cattle (*Bos taurus* and *Bos indicus*), the shape and size of horns have become the prominent feature of their unique phenotypic diversity between the breeds (Ajmone-Marsan *et al.* 2010). Generally, the head phenotype in cattle is termed as *Horned* when keratin coated permanent pointy protrusions anchored to the skull, *Scurred* when rudimentary horns are loosely attached to the skin rather than the skull, or *Polled* when there is a complete absence of horns and scurs. Absence and presence of horns or scurs is determined during prenatal development. However, the postnatal development of horns *vs.* scurs is very difficult to differentiate at an early age because both present initially as free-floating horn-buds and subsequently, only the former fuses to the cranium (Dove 1935; Wiener *et al.* 2015).

Desirability of horns has changed over the centuries with developments in the intensity and practices of raising beef and dairy cattle (Zeder 2008; Ajmone-Marsan *et al.* 2010). In the modern commercial cattle industry, horned animals are less desirable because they pose potential hazards for other cattle and animals used for mustering (horses, dogs), feeding, handling and transport facilities, and farm workers. Furthermore, there is evidence that horned cattle are associated with higher costs of on-farm and post-farm production and greater risk of reduced meat and skin quality (Bunter *et al.* 2013; Knierim *et al.* 2015; Schafberg and Swalve 2015). Therefore, polled cattle have generally become much more desirable. Archaeological evidence suggests that polled cattle have existed in various civilizations for at least a few centuries (Kyselý 2010; Lauwerier 2015; Schafberg and Swalve 2015). A range of different surgical, chemical and cautery methods are used to either remove unattached horn buds (disbudding) or horns attached to the cranium (dehorning). All procedures are costly and cause varying degrees of pain and reduced animal welfare, morbidity, mortality and reduced productivity, with dehorning often resulting in exposure of the frontal sinus (Stafford and Mellor 2005; Neely *et al.* 2014; Knierim *et al.* 2015; Herskin and Nielsen 2018).

Selective breeding for polled animals requires accurate early-in-life prediction of horn phenotype (Spurlock *et al.* 2014; Scheper *et al.* 2016). Horn phenotype is a qualitative trait controlled by genetics – 000483-9913 (OMIA 2019) – which have been mapped to bovine autosome 1 (BTA1) (Georges *et al.* 1993; Harlizius *et al.* 1997; Mariasegaram *et al.* 2012; Randhawa *et al.* 2016). The underlying genes and causal mutations for horns, scurs and polledness remain to be fully elucidated. Phenotypic penetrance suggests that the poll gene is dominantly inherited, *i.e.*, PP (polled), pp (horned), with heterozygous animals usually polled but also commonly scurred (Pp). The genetic basis of scurs remains unclear although evidence suggests the condition is genetically complex and affected by polled status as well as sex of individuals (Capitan *et al.* 2009; Mariasegaram *et al.* 2010; Capitan *et al.* 2011; Tetens *et al.* 2015). In addition, an as yet unidentified African horn gene has been speculated as a possible explanation for the epistasis-like complexity in the horn inheritance in several breeds (White and Ibsen 1936; Long and Gregory 1978; Prayaga 2007). However, no empirical evidence has been presented to confirm its existence to date (Grobler *et al.* 2018).

Genetic heterogeneity has been found across breeds linking the poll characteristics with different sequence variants of deoxyribonucleic acid (DNA), namely, *Celtic* (P_C_), *Friesian* (P_F_), *Mongolian* (P_M_) and *Guarani* (P_G_) mutations (Medugorac *et al.* 2012; Rothammer *et al.* 2014; Wiedemar *et al.* 2014; Medugorac *et al.* 2017; Grobler *et al.* 2018; Utsunomiya *et al.* 2019). Each mutation is a complex insertion-deletion of variable size, such that P_C_ is g.[22429326_2429335del;2429109_2429320dupins], P_F_ is g.2629113_2709240dup (80,128 bp), P_M_ is g.[2695261_2695267delinsTCTGAA;2695889_2696047dupins] and P_G_ is g.2614828_2724315dup (∼110 kb) within the poll locus (2.2 to 2.8 Mb) on BTA1 (bovine genome assembly ARS-UCD1.2 (GCA_002263795.2): https://www.ncbi.nlm.nih.gov/assembly/GCF_002263795.1/). These genetic mutations (P_C_, P_F_, P_M_ and P_G_) are not directly involved in gene coding, although putative causal effects have been reported by introgression of the P_C_ allele by gene-editing of bovine embryos that resulted in the birth of healthy and phenotypical unremarkable polled cattle (Carlson *et al.* 2016). The mutations may be involved in gene regulation and translation processes through unconventional mechanisms as speculated by the presence of antisense sequences caused by similar insertions disturbing normal function of horn growth associated genes (Allais-Bonnet *et al.* 2013; Wiedemar and Drögemüller 2015). However, their association with polledness provides opportunities for genetically selecting animals to produce naturally polled cattle (Prayaga 2007; Spurlock *et al.* 2014; Windig *et al.* 2015). Notably, P_C_ and P_F_ are the most frequent mutations observed in the majority of breeds in production systems globally, and hence are the focus of this study.

The poll locus contains several microsatellite (MSAT) markers, unique to several breeds, that have been successfully employed to predict the poll phenotypes (Mariasegaram *et al.* 2012). However, within-population instability and cross-population diversity of these obsolescent genetic markers make them vulnerable to diminishing accuracy over time and in non-ascertained populations. As genome sequencing technologies have become more accessible and cost effective, SNP-based testing has replaced MSAT testing. SNP-based indirect tests have been developed to predict both P_C_ and P_F_ alleles and are present on many commercial genotyping assays designed for genomic evaluation to reduce testing costs. However, the accuracy of commercially available SNP-based poll gene tests are constrained because their initial development only involved a limited number of taurine cattle breeds (Grobler *et al.* 2018). To address the problem that the current commonly used SNP haplotype translations are not well suited to several beef breeds, including Brahman and Brahman-infused cattle common to northern Australia and other tropical countries, an optimized poll gene test was developed using the recently identified SNPs associated with Celtic and Friesian mutations, and a resource herd with accurate phenotypic and genotypic recording across generations. This paper describes the development and evaluation of this optimized poll gene test for cattle.

## Materials and Methods

### Animal ethics

Ethics approval for the pre-tested animals was not required, as these results were generated under commercial services using microsatellites and SNP based poll testing. However, tail-hair samples, head phenotypes and genotyping for confirmation of the optimized poll gene test were approved (Animal ethics approval numbers SVS/301/18, SVS/465/18 and SVS/ANRFA/397/19).

### Sampling, genomic and phenotypic records

A total of 39,943 cattle samples were used in this study to compare the previously performed diagnostic predictions with the proposed optimization and assess their phenotypic concordance (Table 1, Table S1). The poll predictions previously tested with MSAT (n = 20,534) were obtained from Neogen Australasia. Genomic data and CPT-based predictions consisted of 20,636 samples. Phenotypic information about registered animals in Australia were obtained from the BREEDPLAN database (http://breedplan.une.edu.au/index.php). Tail-hair samples of Brahman (n = 60) were used from previously available stocks for DNA sequencing. In addition, tail-hair samples of validation populations including Droughtmaster (n = 156), Brangus (17), Brahman (22), Santa Gertrudis (29) and cross-bred (33) cattle were collected for phenotypic and genetic concordance evaluation (Table 1).

**Table 1:** Number of animals tested by Microsatellites and SNPs based assays

Breed	Tested animals	Tested by MSAT	Tested by SNP	Validation population
Angus	1630	28	1602	
Brahman	7160	4532	2819	22
Brangus	806	745	72	17
Charolais	3148	2666	900	
Droughtmaster	2718	2223	558	156
Hereford	6424	3485	3341	
Limousin	2193	2124	207	
Santa Gertrudis	4456	4306	136	29
Shorthorn	316	224	67	
Wagyu	9182	201	9050	
Other breeds*^a^*	1910	—	1884	33
Total	39,943	20,534	20,636	257

a List of other breeds are provided in Table S1.

### Microsatellites, SNP markers and current poll testing assays

A total of 14 microsatellite (MSAT) markers located between positions 2,341,080 to 3,014,463 on BTA1 have strong associations with polledness across different populations (Table S2). The poll-haplotype diagnostic test data set in the current study used 10 markers for predictions (Mariasegaram *et al.* 2012; Piper *et al.* 2014). There are over twelve thousand SNPs surrounding the poll locus in cattle, of which only 10 have shown strong association with the candidate mutations (Celtic and Friesian) causing polledness (Table 2) (Medugorac *et al.* 2012; Rothammer *et al.* 2014; Wiedemar *et al.* 2014). Out of the 10 SNPs associated with polledness in various cattle breeds and with strong LD to the known Celtic and Friesian variants (Table 2), the current poll testing (CPT) assay represents 8 SNPs. Within this haplotype, the SNP rs800947704 has been identified as having low call rates in several breeds (Table S3), and therefore was investigated by DNA sequencing.

**Table 2:** List of single nucleotide polymorphisms (SNP) on autosome 1 known for strong linkage disequilibrium (LD) with Celtic (P_C_) and Friesian (P_F_) mutations and their call rate in cattle breeds

SNPs	ARS-UCD1.2 positions	Mutations	Poll alleles	LD with	Call rate *^a^*
rs801127025	2,372,456	P_5ID_	T	P_F_	99.47
rs799187101	2,377,687	G > A	A	P_F_	99.98
rs800947704	2,378,745	C > T	T	P_F_	96.32
rs798116945	2,407,338	G > C	C	P_F_	99.99
rs383143898	2,429,319	P_202ID_	T	P_C_	99.92
rs799403053	2,486,811	T > C	C	P_F_	99.80
rs210350155	2,491,161	C > A	A	P_F_	99.23
rs797088784	2,578,598	G > A	A	P_F_	99.44
rs800767839	2,629,115	T > A	A	P_F_	99.94
rs799920960	2,748,715	C > G	G	P_F_	100

a indicates mean call rate. Breed-wise call rates are given in Table S3.

### DNA extraction, PCR, DNA sequencing and SNP genotyping

DNA extraction from all samples used for targeted DNA sequencing and SNP genotyping was performed by using the standardized protocol at the genetic testing laboratory of Neogen Australasia. DNA fragments of 1,098 bp (2,377,810-2,378,907) harboring a targeted genotype rs800947704 (g.2378745G > C) were amplified by using primers (Forward: 5′-TCCCTCTGCTGTGATAAACACC-3′ and Reverse: 5′-ACCACAAACCAAGGCCAAAC-3′). PCR reactions containing 15-20 ng DNA, 10 μM forward and reverse primers, 0.12 μl taq in a total volume of 25 μl, and employed standard PCR cycling conditions on an Applied Biosystems thermocycler. An internal primer (5′-TCCAATGAACACCCAGGACT-3′) was used to generate 665 bp (2,378,243-2,378,907) sequencing reads. Unused dNTPs and primers were removed using ExoSAP-IT (USB Corporation distributed by GE Healthcare Bio-Sciences, Rydalmere, Australia). Sequencing was performed using an ABI Prism Big Dye Terminator Cycle Sequencing Ready Reaction Kit Version 3.1 (PE Applied Biosystems, Foster City, USA) following the instructions supplied with the kit. Sequencing separation was performed on an ABI 3730xl automated sequencer. Forward and reverse sequences were aligned and edited using ChromasPro (Technelysium Pty Ltd, Tewantin, Australia).

#### Prediction of celtic and friesian alleles:

Prediction of polledness-associated P_C_ and P_F_ mutations were generated using the SNP markers (Table 2) available on commercial bovine BeadChip assays (Illumina) including Neogen’s proprietary GGP-LDv4, GGP Taurus 50K or GGP Indicus 35K assays (Neogen Corporation, Lincoln, NE). The Celtic (P_C_) is predicted by translating a single SNP marker rs383143898 based on its horn or poll allele (Table 2). The Friesian mutation is predicted based upon haplotype associated with markers in LD with P_F_ (Table 2). Note that the combination of P_F_-markers used varied with different versions of the Poll Test being assessed in this study. Final results represent reconciled outcomes from both predictions to generate allele-pairs (genotypes) such as HH, HP_C_, HP_F_ P_C_P_C,_ P_C_P_F_ or P_F_P_F_. However, if the P_C_-SNP or more than two P_F_-SNPs fail during genotyping, or two or more SNPs differ in predicted genotype (H *vs.* P_F_) then the result is considered ambiguous and termed as a “No Result”. The optimized poll testing assay remained identical for P_C_, while genotyping failure or contrasting prediction were restricted to only one differing or missing P_F_ SNP.

### Data availability

Genomic sequences generated in this study have been deposited in NCBI’s GenBank (accession numbers: MN473394 to MN473448). Genotypic and phenotypic data have been provided in supplementary files Data 1 and Data 2 available at figshare: https://doi.org/10.25387/g3.10423904.

## Results and Discussion

Distinctive characteristics of MSAT and SNP in different populations have historically made interpretation of the poll gene test quite complex, resulting in compromised performance of both test types in some breeds. Out of 20,534 samples tested with the MSAT-based assays, 11.7% have failed to predict a poll genotype - reported as “Not Determined” (Figure S1). Breed specific failure varied from Angus (3.57%) to Wagyu (30.35%), while most breeds have the failure rates over 10% (Brahman, Brangus, Charolais, Droughtmaster, Santa Gertrudis, Shorthorn). The high frequency of Not Determined was a major barrier to widespread uptake of the MSAT-based poll test in Australia.

SNP-based poll testing in cattle has significantly reduced the frequency of Not Determined (No Result) in taurine breeds. However Zebu (*Bos indicus*) and their cross-bred cattle are still constrained by a high frequency of No Results (Figure S1). Out of 20,636 samples genotyped with the current SNP-based poll test (CPT), most of the breeds of European origin (Angus, Charolais, Hereford, Limousin, Holstein-Friesian etc.) except Shorthorn were successfully predicted for head-status (Table S1). Nonetheless, overall 9.69% of CPT-tested samples had failed to identify an unambiguous genotype and hence were predicted as “No Result” with the majority of these No Results being Brahman (17.45%) and Brahman-infused populations such as Brangus, Droughtmaster, Santa Gertrudis and Cross-bred cattle (Table S1, Figure S1).

We investigated the utility of the 10 SNPs that make up the CPT assays in the predictions of P_C_ and P_F_ prevalence in different breeds. Two SNPs, rs798116945 and rs800767839, were homozygous across most of the European and Zebu breeds and their cross-bred populations. Hence, these two SNPs were declared non-informative for the Poll gene testing assays (Table 2, Table S3). Our investigation also identified two SNPs, rs799187101 and rs800947704, as the major cause of “No Result” in the Zebu cattle when running the CPT assay Neither SNP is in complete LD in the haplotypes predicting the presence of P_C_ or P_F_. Furthermore, in a subset of 18,675 genotyped animals, SNP rs800947704 consistently showed lower call rate (Table S3) in several breeds including Brahman (14.3%). Therefore, 60 Brahman samples were selected to sequence the 665 bp genomic region surrounding rs800947704 in 55 samples (Table S4, GenBank accession numbers: MN473394 to MN473448). Sequence alignment of 55 Brahman samples found nine known SNPs (rs383371521, rs377981008, rs135217384, rs136702754, rs134535435, rs381418143, rs800947704, rs110759734 and rs444879378).

Targeted sequencing analyses of Brahman cattle suggested that there was an inherent issue with the probe design of rs800947704 SNP in the various Illumina BeadChips (Table S4). Genotype call failure at the target SNP (C > T, rs800947704) in the genotyping assays was being caused by probe hybridization issues, due to a neighboring SNP rs381418143 (g.2378742A > G) located 3 bp upstream to rs800947704 in Brahman cattle. The current probes are designed based upon the Taurine reference genome to recognize allele A only of rs381418143. Therefore all samples carrying the allele A at rs381418143 (n = 22) were correctly genotyped at the target SNP rs800947704 for all alleles (CC, TT or CT = Y). However, DNA samples (n = 16) with G at rs381418143 resulted in incorrect genotype or failure to generate a genotype depending upon whether animals tested were heterozygous GA or homozygous AA for rs800947704. Although some of the genotyping assays were optimized to fix the aforementioned errors, it was observed that the poll-associated allele T of rs800947704 was not consistently genotyped in samples when other marker SNPs were indicating the presence of P_F_ associated alleles. Similarly, another SNP rs799187101 was found discordant causing unsuccessful predictions for P_F_. Therefore, both SNPs (rs799187101 and rs800947704) were deemed unreliable and excluded. Finally, SNP rs799920960 was found to be redundant and was also excluded.

At this point, the remaining 5 SNPs (rs801127025, rs383143898, rs799403053, rs210350155 and rs797088784) were retained to assess accurate predictions of P_C_ and P_F_ alleles, with rs383143898 (P_202ID_) as the sole predictor for P_C_, while the other 4 SNPs are associated with P_F_. For the sake of this prediction we accepted the successful genotyping of at least 3 out of the 4 SNPs as minimum to predict P_F_. Collectively, these 5 SNP markers are named as optimized poll testing (OPT) assay, which predicts 6 possible genotypes (HH, HP_C_, HP_F_, P_C_P_C_, P_C_P_F_ or P_F_P_F_). We evaluated previously successful predictions (n = 18,637), confirming the predictions remain unchanged (100%) using OPT relative to the original prediction with CPT (Table 3). Of the samples previously unable to be predicted based upon the CPT translations (No Result, n = 1,999), 1,990 (99.6%) were effectively classified into one of the 6 genotypic predictions (Table 3, Table S1). Thus, out of the total genotyped samples (n = 20,636) the success rate for OPT predictions was 99.96% as compared to 90.31% for the CPT assays. Subsequent investigation of the remaining unpredicted (No Result) 9 samples found that 8 of them were due to genotyping failure for rs383143898 (P_C_) and one failed for multiple markers associated with P_F_ (Table S1). Both genotyping error rates were within the expected < 0.01% range (Wu *et al.* 2019) and can be resolved by resampling. It is possible that higher prediction failure rates by MSAT and CPT in some breeds (*e.g.*, Brahman and Wagyu) might suggest that they carry another mutation (*e.g.*, Mongolian) given the spatial and temporal closeness with Turano cattle. However, 100% of polled animals of these breeds in this study carried P_C_ or P_F_ as elucidated by the OPT. Hence, we conclude that with successfully genotyped OPT markers, 100% of the samples can be effectively predicted in multiple breeds of cattle including European, Zebu and their composite animals (Table S1, Figure S2).

**Table 3:** Comparison of current (CPT) and optimized (OPT) poll testing on 20,636 Australian cattle samples. List of breed-wise No results is given in Table S1

Animal genotypes	CPT results	OPT results	Gain by OPT
HH	11,113	12,785	1,672
HP_C_	3,420	3,696	276
HP_F_	135	147	12
P_C_P_C_	3,641	3,651	10
P_C_P_F_	300	311	11
P_F_P_F_	28	37	9
No Result	1,999	9	−1,990

Accuracy of true phenotype matching with predicted genotypes is challenging because of a lack of phenotypic records as well as inaccuracies (Connors *et al.* 2018). Phenotypic records on a subset of 6,930 animals were obtained from the BREEDPLAN database to test concordance between phenotypes and genotypes (Table 4). OPT-based genotypes have shown high concordance with known head-status for HH and PP animals. However the potential for *scur* phenotypes in heterozygous animals (HP_C_ and HP_F_) significantly compromises informativeness of these genotypes. The biology underpinning scur development remains poorly understood and potentially multifactorial, with indications in literature pointing to the probability that gender (female) and sex hormones (steer) sway heterozygotes to be *poll* (Pailhoux *et al.* 2001). It is very unlikely that HH animals can be either *scurred* (3.1%) or *polled* (2.1%), and these percentages likely reflect errors due to imprecise phenotyping at a very early age, improper dehorning resulting in partial regrowth of horns mimicking scurs and data recording errors. Accuracy of phenotype recording remains a significant challenge as evident in the BREEDPLAN data set. In the validation data, phenotype-genotype concordance was found as expected with HH (100% horned) and P_C_P_C_ / P_C_P_F_ / P_F_P_F_ (100% polled) except HP_C_ (59.9% scurred and 40.1% polled) and HP_F_ (100% polled). If in the future true discordance between genotypes and phenotypes is confirmed, *e.g.*, P_C_P_C_ being horned or HH being polled, these animals should be considered be a valuable resource for further study. We have noted that heterozygous animals carrying P_F_ (*i.e.*, HP_F_) were significantly (Fisher’s exact test, *P* = 0.05) more likely to be polled compared to HP_C_. This observation may warrant further study in the future as the numbers of HP_F_ genotypes is too small (n = 123) in both data sets (Table 4). On the other hand, a larger number of P_C_P_F_ animals (n = 269) were found to be 99.3% polled (Table 4). Note that P_C_ and P_F_ mutations are approximately 200 kb apart and expected to be *in trans*-arrangements given their independent evolutions. This study have found no occurrence of *cis*-gametes carrying P_C_ and P_F_ on the same haplotype in P_C_P_F,_ P_F_P_F_ and P_C_P_C_ animals (n = 7,640, Table 3). However, there is non-negligible recombination probability (∼0.2%) and a P_C_P_F_ *cis*-haplotype coexisting with H allele on second haplotype may cause growth of scurs as unexpected phenotype. To date it remains unclear whether the existence of a scur gene could be involved in the diversity of scurred animals as well as polled phenotypes in heterozygotes HP_C_ and HP_F_ (Tetens *et al.* 2015). We continue to investigate further genetic factors in target populations by using high-density SNP arrays, whole genome sequencing and accurate phenotyping.

**Table 4:** Phenotypic concordance (%) between OPT genotypes and BREEDPLAN and validation samples

OPT genotypes	Concordance with BREEDPLAN				Concordance in validation population			
	N	Horn	Scur	Poll	N	Horn	Scur	Poll
HH	2,800	94.8%	3.10%	2.10%	66	100%	—	—
HP_C_	2,121	13.5%	15.7%	70.8%	137	—	59.9%	40.1%
HP_F_	120	2.50%	5.80%	91.7%	3	—	—	100%
P_C_P_C_	1,595	0.75%	0.25%	99.0%	49	—	—	100%
P_C_P_F_	267	0.37%	0.37%	99.2%	2	—	—	100%
P_F_P_F_	27	—	—	100%	0	—	—	—

Economic sustainability and animal welfare have driven recent progress in modern livestock production systems especially in efforts to minimize or eliminate undesirable traits such as the presence of horns in cattle. Genetic dehorning is being progressively adopted as the non-invasive approach to breed hornless cattle through genetic selection (Carlson *et al.* 2016; Mueller *et al.* 2019). Genotype-phenotype relationships of horn growth are however complex (Medugorac *et al.* 2012) limiting the informativeness of current assays for early detection for horn status, and the presence or absence of polled alleles (polledness), in some breeds. In this study we identified limitations of the current poll gene testing assays, especially in tropical cattle common in Northern Australia and throughout Asia. Our investigations have demonstrated that a robust set of 5 SNPs can effectively eliminate the ambiguous and undetermined results that limited the effectiveness of previous poll predictions. The next important step is to get a better understanding of the Scur locus, which remains a confounding influence especially for heterozygous (HP_C_, HP_F_) animals.
